# Prediction of organic homolytic bond dissociation enthalpies at near chemical accuracy with sub-second computational cost

**DOI:** 10.1038/s41467-020-16201-z

**Published:** 2020-05-11

**Authors:** Peter C. St. John, Yanfei Guan, Yeonjoon Kim, Seonah Kim, Robert S. Paton

**Affiliations:** 1 Biosciences Center, National Renewable Energy Laboratory, 15103 Denver West Parkway, Golden, CO 80401 USA; 2 Department of Chemistry, Colorado State University, Fort Collins, Colorado 80523 USA; 3 Chemical Research Laboratory, University of Oxford, Mansfield Road, Oxford, OX1 3TA UK; 4 Present Address: Department of Chemical Engineering, Massachusetts Institute of Technology, 77 Massachusetts Ave., Cambridge, MA 02139 USA

**Keywords:** Cheminformatics, Thermodynamics, Computational chemistry

## Abstract

Bond dissociation enthalpies (BDEs) of organic molecules play a fundamental role in determining chemical reactivity and selectivity. However, BDE computations at sufficiently high levels of quantum mechanical theory require substantial computing resources. In this paper, we develop a machine learning model capable of accurately predicting BDEs for organic molecules in a fraction of a second. We perform automated density functional theory (DFT) calculations at the M06-2X/def2-TZVP level of theory for 42,577 small organic molecules, resulting in 290,664 BDEs. A graph neural network trained on a subset of these results achieves a mean absolute error of 0.58 kcal mol^−1^ (vs DFT) for BDEs of unseen molecules. We further demonstrate the model on two applications: first, we rapidly and accurately predict major sites of hydrogen abstraction in the metabolism of drug-like molecules, and second, we determine the dominant molecular fragmentation pathways during soot formation.

## Abstract (web summary)

Bond dissociation enthalpies are key quantities in determining chemical reactivity, their computations with quantum mechanical methods being highly demanding. Here the authors develop a machine learning approach to calculate accurate dissociation enthalpies for organic molecules with sub-second computational cost.

## Introduction

Nearly all chemical reactions of organic compounds involve the breaking and formation of covalent bonds. Unsurprisingly, bond energies feature as an essential ingredient in many predictive models of chemical reactivity. Homolytic bond dissociation enthalpies (BDEs) are defined by the enthalpy change for the gas-phase reaction at 298 K:

1 $$A - B \to A \cdot + B \cdot$$

The cumulative difference between BDE values of all bonds broken and formed in a chemical reaction thus provides an estimate of the overall reaction enthalpy^1^. BDE values are thermodynamic quantities but they are also used widely to predict reaction kinetics. For example, BDE values are used to predict relative reaction rates using well-established Evans–Polanyi-type correlations with bond strengths in radical hydrogen atom abstractions^2^. BDEs also provide insight into thermodynamically accessible reaction mechanisms for a given compound, and their calculation is often the first step in characterizing dominant pathways in combustion^3^, polymer synthesis^4^ and thermal stability^5,6^, lignin depolymerization^7^, drug metabolism^8–10^, explosives^11^, organic synthesis planning^12,13^, and other applications to energy-related materials^14^.

The accurate measurement and calculation of BDEs underlies numerous applications in organic chemistry. Experimental measurement of BDEs for polyatomic molecules are difficult, but a variety of techniques exist^15^ with a typical uncertainty of ±1–2 kcal mol^−1^^16^. Calculation of BDEs with ab initio quantum chemistry methods is possible, however, the choice of method is known to greatly affect the resulting computational accuracy^17^. Despite this, density functional theory (DFT) computations using M06-2X and M05-2X functionals have been shown to achieve accuracies comparable to the uncertainties of the underlying experimental measurements^18^. As a result, quantum mechanical (QM) methods play an integral role in calculating radical enthalpies and proposing reaction mechanisms. However, even relatively efficient QM methods such as DFT scale exponentially with basis set size, often taking hours or days to obtain a single BDE value. This conventional workflow requires the geometry of a reactant and its radical products to be optimized and the Hessian of each species evaluated. For flexible compounds this process must be repeated for several alternative conformations. The integration of BDE calculations in molecular design efforts, including quantitative structure–property relationship (QSPR) models, has thus been limited by these computational demands, and the use of BDE calculations for the screening of thousands or millions of candidate structures remains impractical. In this manuscript we describe a new computational workflow that overcomes these limitations.

The rise of machine learning (ML) in quantum chemistry has led to the development of highly-accurate empirical models^19^ that have accelerated traditionally difficult QM calculations for predicting enthalpy^20^, optoelectronic properties^21^, and forces^22^. In particular, the rise of graph neural networks (GNNs)^23^ in modeling chemical properties has enabled ‘end-to-end’ learning on molecular structure: a ML strategy where traditional feature engineering is replaced by feature learning from a graph-based molecular representation^19^. These approaches have led to best-in-class prediction accuracies on a range of applications, especially as the amount of available training data grows^24,25^. An open question in molecular machine learning is whether optimized 3D coordinates are required as inputs to the ML algorithm to reach optimal accuracies. For enthalpy prediction on the QM9 dataset, consisting of all small molecules satisfying known valence rules, 3D coordinates appear to lead to superior prediction performance^20^. However, a recent study has shown that for some molecules and properties, 3D coordinates did not necessarily lead to improved results over more simple representations of 2D connectivity and atom types (i.e., SMILES^26^ notation)^21^. In addition, while precise, absolute QM-derived atomization energies are often inaccurate by up to a full Hartree for common molecules (627 kcal mol^−1^)^27^. Direct prediction of reaction energies may therefore be more reliable when compared with experimental values.

**Fig. 1:**
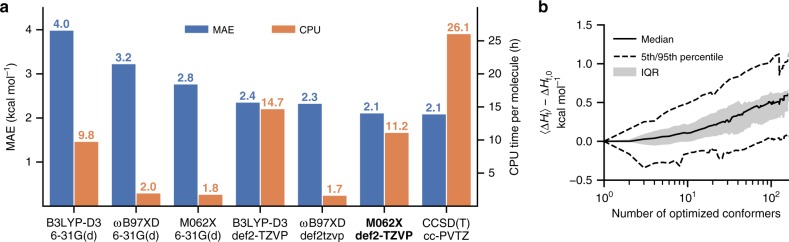
Benchmark study of DFT methods. **a** Trade-off between accuracy (left axis, blue) and computational cost (right axis, orange) for a selection of common QM methods. M06-2X/def2-TZVP was selected for subsequent calculations. MAE and CPU time were averaged over 368 different bonds. **b** Effect of conformer sampling. Molecules were optimized with MMFF94s, and the lowest-energy conformers were used to initialize DFT calculations. The plot shows the difference between the Boltzmann average enthalpy for the entire ensemble and the DFT-calculated enthalpy of the first conformer as a function of the number of optimizations performed. Exhaustive conformer sampling only changes the median resulting enthalpies by <0.5 kcal mol^−1^, with a relatively narrow inner-quartile range (IQR).

For the prediction of BDEs, a previous study leveraged >12,000 DFT calculations and an associative neural network to achieve a mean absolute error (MAE) of 3.4 kcal mol^−1^ for unseen bonds relative to DFT results^28^. This model is based on fixed molecular descriptors calculated for each target bond, and thus does not allow the model to learn more detailed descriptions of each bond as more molecular structures and data is added. B3LYP values were used to train this model, however, this functional poorly captures the enthalpies of radical reactions^29^. In our own benchmarking studies this level of theory has an average error 2 kcal mol^−1^ larger than other DFT methods against experimental BDE values (see below, Fig. 1a). Other existing work has used neural networks to predict the contribution of each bond to the overall atomization energy of closed-shell molecules without explicitly calculating radical enthalpies^30^. While this technique reproduces general trends in overall bond strength, quantitative comparison with experimental BDEs results in MAEs of ~10 kcal mol^−1^. More generally, the use of atomization energies as a benchmark for ML algorithms does not guarantee accuracy in predicting more chemically-relevant reaction energies^31,32^. The development of an accurate ML pipeline to quickly estimate BDEs, with acceptable accuracy compared with experimental values, thus remains a challenge.

In this study, we develop A machine-Learning derived, Fast, Accurate Bond dissociation Enthalpy Tool (ALFABET) to predict homolytic BDEs at close to chemical accuracy with sub-second computational cost. To accomplish this, we first benchmark several quantum chemistry methods on a database of experimentally measured BDEs^33^, finding that the M06-2X/def2-TZVP level of theory has the optimal trade-off between empirical accuracy and computational efficiency. A database of 42,577 closed-shell compounds with nine or fewer heavy atoms and consisting only of C, H, O, and N atoms is then curated from PubChem^34^. Each single bond in the database that was not present in a ring is cleaved to yield two open-shell radicals. DFT enthalpy calculations are then performed on all open and closed-shell molecules to yield 290,664 unique BDEs, representing over 80 days of total CPU time. We then train a graph neural network on a subset of these results, achieving a MAE of 0.58 kcal mol^−1^ when predicting BDEs for unseen closed-shell molecules (compared with DFT results). When compared against experimental values for large molecules not included in the training set, the ML method adds only 1 kcal mol^−1^ to the MAE of the DFT approach, while completing in less than a second (compared with over a day per molecule for DFT). The utility of the developed prediction tool is subsequently demonstrated on two separate applications where fast, accurate prediction of the weakest bond in a molecule is required. First, the model is used to rapidly and accurately predict the site of C–H oxidative degradation in large, drug-like molecules. The model replicates the results of much more expensive DFT calculations with an MAE of 1.14 kcal mol^−1^, and 95% of metabolic sites occur at bonds within 2 kcal mol^−1^ of the weakest bond in the molecule. Second, the model is used to predict the dominant radicals formed during combustion of fuel molecules, and the identities of these radicals are used as features for a QSPR model of soot formation pathways. These applications demonstrate the broad applicability of the developed tool and demonstrate that bond strength prediction for organic molecules can be reliably performed using fast ML techniques.

## Results

### Evaluation of QM methods for calculating homolytic BDEs

In order to ensure that the resulting ML method closely reproduced experimentally determined BDEs, we performed a benchmark study of common DFT and ab initio methods. Computed gas-phase BDE values include unscaled vibrational zero-point energies and thermal corrections to the enthalpy at 298 K and 1 atm, using optimized geometries obtained following a conformational search (see below). For a set of 368 experimentally measured BDEs from the *iBond* database^33^, combinations of three different DFT functionals (B3LYP-D3^35,36^, ωB97XD^37^, and M06-2X^38^) and two basis sets (6-31G(d) and def2-TZVP) were compared with DLPNO-CCSD(T)/cc-pVTZ calculations (Fig. 1a). As expected, the CCSD(T) calculations took the longest to perform and were the most accurate. Of the DFT methods, the choice of basis set appeared to have the greatest impact on accuracy, with the M06-2X/def2-TZVP combination coming very close to CCSD(T) accuracy. MAEs of the three density functionals followed the order of B3LYP-D3 > ωB97XD > M06-2X for both basis sets. This is consistent with previous benchmarks against the stabilization energy of 43 radical species calculated using CCSD(T)/CBS^31,39,40^. The observed MAE of top performing methods approaches the underlying uncertainty in the experimental measurements.

Conformer sampling was performed using the RDKit library^41^, using the MMFF94s force field^42^. Between 100 and 1000 conformers were generated for each molecule, depending on the number of rotatable bonds. The lowest-energy conformer identified by force-field calculations was then used as an initial guess for subsequent geometry optimization at the higher level of theory. For radicals, initial structures were generated by temporarily replacing the radical with a bonded H atom during force field optimizations. The enthalpy of formation of this first conformer was denoted $\Delta H_{f,0}$. As a reordering of conformational energies often occurs upon reoptimizing MM geometries with a higher level of theory, we analyzed the typical error introduced by only optimizing the MM global minimum energy conformer at the higher level of theory. By optimizing additional higher-energy (i.e., local minima) MM conformers we can calculate the difference between our initial enthalpy estimation, $\Delta H_{f,0}$, and the Boltzmann-weighted enthalpy (at 298 K) of the entire conformer ensemble, $\ \langle {\Delta H_f} \rangle$. The difference between these quantities is plotted in Fig. 1b, indicating that the median error introduced by only optimizing a single conformer (versus an ensemble of over 100) is only ~0.5 kcal mol^−1^, while requiring 1/100th the computational resources. We therefore proceeded with database construction at the M06-2X/def2-TZVP level of theory and the computational pipeline described above (and in more detail in the methods section), optimizing only the most stable MM conformer.

### Construction of a machine-learning compatible BDE database

We next developed a large database of BDE values, BDE-db, on which to train ALFABET. To maximize the variety of bond strengths for a minimum computational effort, we limited the initial database construction to molecules with 10 or fewer heavy atoms. In addition, smaller molecules reduce the risk of the geometry optimization finding a local energy minimum substantially higher than the true global minimum.

**Fig. 2:**
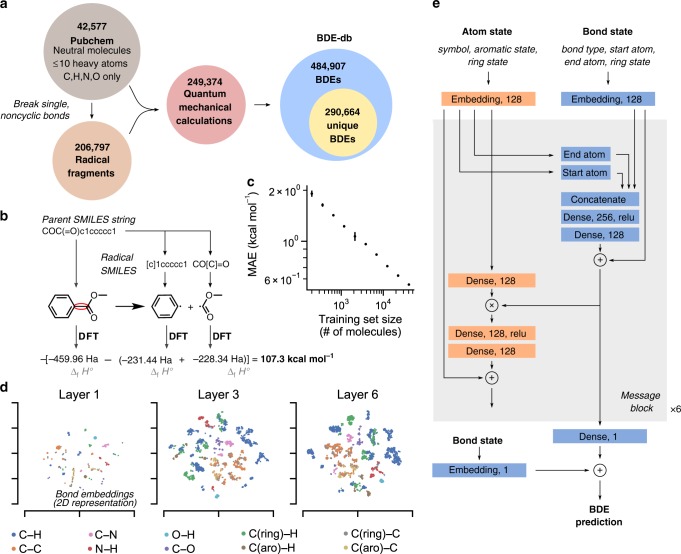
Overview of database construction and GNN structure. **a** Size of key elements of BDE-db. **b** Indexing and calculation of a single BDE reaction. For a given cleaved bond, SMILES strings of the parent molecule and two resulting radicals are passed for DFT optimization. **c** Learning curve for the model, plotting MAE (in kcal mol^−1^) on dev set BDEs against the number of molecules included in the training database. Both *x* and *y* axes are log-scaled, and error bars indicate standard deviation between three replicates. **d** 2D representations of bond embeddings are shown via the t-SNE algorithm after the first, third, and final message passing layers. Initially, bonds of similar classes are clustered close together in embedding space. For deeper layers of the model, representations of the bonds become more detailed as they represent its specific local environment. **e** Structure of the GNN. Atom and bond state vectors are updated through a series of six message passing blocks. The final embedding layer is then used to predict the BDE of each bond.

Construction of BDE-db began with 42,557 parent C_x_H_y_O_z_N_m_ molecules taken from the PubChem Compound database (Fig. 2a). Only neutral molecules with assigned CAS numbers were used during database construction. Each single, non-cyclic bond in these molecules was then cleaved to generate two child radicals which were also added to the database. Canonicalized SMILES strings with specified configuration at stereogenic centers were used to represent these molecules and remove duplicates (Fig. 2b). Child radicals were frequently the product of multiple BDE reactions, reducing the number of DFT calculations required. However, this use of the SMILES language presents some complications for database construction. Specifically, bond cleavage occurring within an enantiotopic or diastereotopic group (that are not differentiated by SMILES) forms radicals with a new and unspecified stereocenter in relation to the parent molecule. The creation of new diastereomeric relationships in the products gives rise to non-equivalent BDE values dependent upon the choice of relative configuration. Dissociations resulting in a new stereocenter were omitted from the database.

DFT calculations were then performed for the parent molecules and unique child radicals. A variety of convergence checks were performed to ensure the DFT optimization converged to a stable structure, including checks for imaginary frequencies and ensuring that the molecule did not further decompose into disconnected molecules (e.g., radical fragmentation of an alkoxyacyl radical into an alkyl radical by loss of CO_2_) or suffer an intramolecular rearrangement (e.g., by a [1,*n*]-H shift). Approximately 10% of attempted DFT calculations were discarded, primarily due to imaginary frequencies. A total of 249,374 successful calculations were used to build the BDE-db. These calculations resulted in 484,907 total calculated BDEs, of which 290,664 were unique (methane has only one unique BDE value). These numbers highlight the efficiency gains achieved through calculating a large database in parallel and reusing calculation results for child radicals, as typically three QM calculations are required per one BDE.

### Development of a graph neural network for predicting BDE

A graph neural network (GNN) was developed to predict BDE directly from molecular structure. GNNs in the past have been used to predict the enthalpy of molecules from their optimized 3D structure, with MAEs close to 0.3 kcal mol^−1^^22^. The application of this technique for the proposed target would require optimized 3D structures of both the parent molecule and child radicals, and prediction errors would likely compound when summing together three separate predictions. We instead sought to develop a model that only required the 2D structure (i.e., SMILES string) of the parent molecule as input. SMILES strings were converted to a graph representation using RDKit (with atoms as nodes and bonds as edges). Each bond in the molecule was represented by two directional edges, pointing in reverse directions between the two bonded atoms.

GNNs operate by mixing information between neighboring nodes and edges. By iteratively updating node and edge internal states depending on the internal states of their neighbors, embedding vectors are generated that serve as a finite-dimensional description of each atom or bond’s local environment (Fig. 2d). For BDE prediction, bond embedding vectors at the final layer are reduced through a linear layer to predict the BDE (predictions from both the forward and backward bond edge are averaged together). The overall network structure was inspired by a model from Jørgensen et al.^43^, but with a simplified interaction structure. As only 2D inputs are used, atom and bond vectors are initialized with embedding layers based on a number of properties inferred via RDKit (Fig. 2e). In each message passing layer, bond states are first updated with information from neighboring atoms, and atom states are then updated with information from neighboring bonds. Residual connections were used for each message passing layer in order to aid convergence of deeper models^44^. Six message passing layers were used in the final model, as no improvement in accuracy was seen for additional layers. The final model structure contains 1.06 M parameters. Bond states from the final message passing layer are reduced to a single BDE prediction by passing them through a linear layer. Following SchNet^22^, these predictions were added to a single mean BDE value for each bond class to generate the final prediction. BDE predictions are therefore generated simultaneously for each bond in the input molecule.

**Fig. 3:**
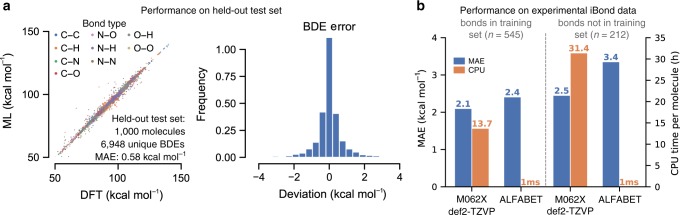
Performance of the ML BDE prediction algorithm. **a** Performance on the held-out, DFT-generated test set. (left) Parity plot of ALFABET predictions vs DFT calculations. BDE points are colored by their bond type. (right) Histogram of prediction errors. The model achieves an MAE of 0.58 kcal mol^−1^ relative to DFT-calculated values of unseen molecules. **b** Performance of the model on experimentally measured BDEs from the *iBond* database. Prediction accuracy was quantified separately for bonds inside the training database (left) and those outside it (right). Molecules and bonds outside the training set tended to be much larger, thus resulting in larger DFT error and long DFT computational times.

Validation (dev) and test sets were each constructed from all BDEs associated with 1000 parent molecules. The training set thus consisted of 40,577 unique parent molecules and 276,717 unique BDEs. A learning curve for the model, comparing performance against the 1000 molecule dev set while varying the number of molecules in the training set, shows a linear log–log relationship (Fig. 2c). This trend suggests that model accuracies could be further improved through the collection of additional BDE data. Performance of the final model was tested against the held-out test set, consisting of 6948 unique BDEs. The MAE on these bonds was 0.58 kcal mol^−1^ (vs DFT), with 95% of predictions falling within 2.25 kcal mol^−1^ of their DFT-calculated values (Fig. 3a). A breakdown of the model’s performance on each individual bond type is shown in Table 1. Since the goal of the method is ultimately to reproduce experimental BDE measurements, the speed and accuracy of the GNN in predicting experimental BDEs from the iBond database was compared with similar predictions generated via the DFT method (Fig. 3b, Supplementary Data 1). For molecules that were a part of the training set, the ML method achieves prediction accuracies versus experimental measurements that rival those of the DFT approach (2.4 kcal mol^−1^ for ML, 2.1 kcal mol^−1^ for DFT). These results compare favorably with previous ML predictions of BDE (Supplementary Fig. 1). However, a more difficult test of the ML approach is for molecules larger than 10 heavy atoms that were not a part of the training database. For these larger molecules, typical DFT calculations required more than a day per molecule. However, the accuracy of the ML method remained acceptable, adding <1 kcal mol^−1^ to the MAE of the DFT method (3.4 kcal mol^−1^ for ML, 2.5 kcal mol^−1^ for DFT) when compared against experimentally measured BDEs. For these molecules, ALFABET was able to predict BDEs for all the bonds in the molecule in under 1 ms per molecule.

**Table 1:** MAEs and counts for bonds in the training and test databases.

Bond type	MAE (train)	Count (train)	MAE (test)	Count (test)
C–H	0.20	306,404	0.52	7735
C–C	0.22	67,822	0.45	1679
N–H	0.35	25,981	1.02	687
C–N	0.31	23,493	0.80	594
C–O	0.33	23,243	0.78	546
O–H	0.44	11,306	1.04	290
N–O	0.47	1557	0.64	43
N–N	0.56	1528	1.14	38
O–O	0.56	283	0.96	10

MAEs comparing DFT-calculated BDEs to ML predictions are shown along with the number of bonds for which the error was computed. MAEs are in kcal mol^−1^.

### Analysis of ALFABET prediction outliers

**Fig. 4:**
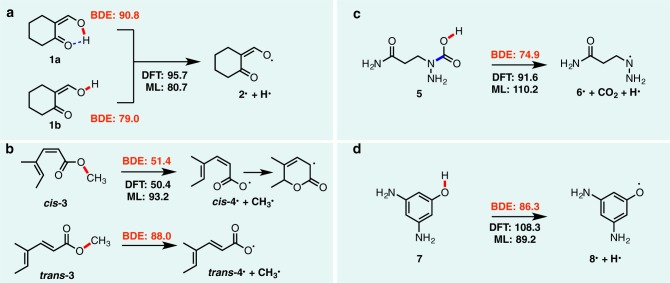
Error analysis of predicted and DFT-calculated BDE values. Ground-truth G4 values (in kcal mol^−1^) for representative molecules with large prediction errors are shown in red.

During construction of BDE-db and ALFABET, we conducted error analyses of preliminary data and models to refine the GNN structure and correct common DFT errors. In this section, we present a more extensive analysis of the remaining large prediction errors (>10 kcal mol^−1^) for bonds in the training, validation, and test sets (Fig. 4, Supplementary Table 1, Supplementary Data 2). In evaluating errors in DFT and ML calculations, additional BDE calculations were performed at the composite G4 level of theory to serve as a ground-truth reference^45^. G4 radical formation enthalpies lie close to experimental values (4.5–6.2 kJ mol^−1^), albeit at an increased computational cost relative to DFT^39^.

ML predictions using deep neural networks have been criticized as being black-box in nature. However, in this study we use the bond embedding vectors from the final message passing layer to interpret the ALFABET predictions, generating a quantitative similarity score to bonds contained in the training database (see methods). These embeddings are subsequently reduced to a single BDE prediction, and thus neighboring bond BDEs indicate how the GNN interprets the input molecule. We found that significant errors can arise in either DFT reference data or the ALFABET predictions due to several recurring structural motifs. In this section, we present examples of several classes of errors that lead to disagreement between DFT calculations and predicted BDEs.

**Fig. 5:**
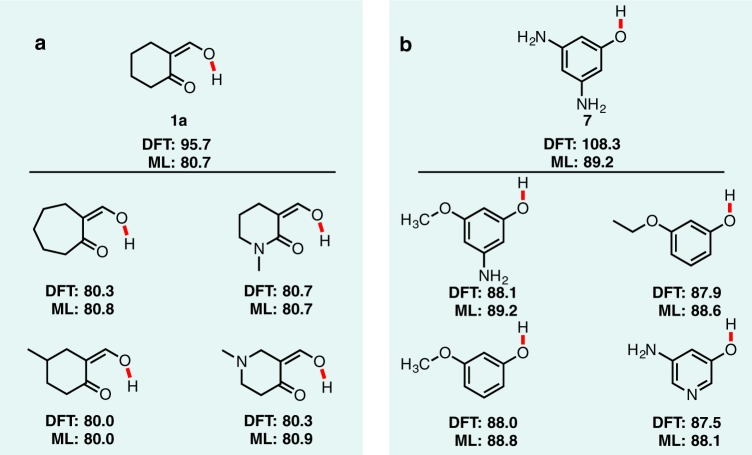
Comparison of outlier calculations to training data. Similar bonds from the BDE-db database for two query bonds (top) from the BDE prediction outliers. **a** Bond from Fig. 4a (**1a**). **b** Bond from Fig. 4d (**7**).

The loss of stabilizing non-covalent interactions such as intramolecular hydrogen bonds by bond dissociation result in prediction errors (Fig. 4a). Relative to the internally H-bonded conformer **1a**, the G4 BDE value is 90.8 kcal mol^−1^. Our DFT reference value was correctly generated using this more stable conformation. However, ALFABET underpredicts this C–H bond strength by 15 kcal mol^−1^, and is much closer to the hypothetical BDE value of 79.0 kcal mol^−1^ for the less stable conformer (**1b**) lacking an H-bond. We can attribute this prediction error to a failure to account for this strong H-bond in the parent compound. Inspection of nearest neighbor structures in the training database (including a similar bond for a 7-membered cycloheptanone) confirm this to be the case, since optimized structures for these molecules lacked internal H-bonds and have DFT values in the ~80 kcal mol^−1^ range (Fig. 5a). For molecules where an intermolecular H-bond is lost or disrupted upon bond cleavage, predictions will tend to underestimate the true BDE value.

Conversely, the development of new stabilizing interactions in radical products result in anomalously low BDE values that are overestimated by ALFABET predictions (Fig. 4b). For example, the carboxyl radical formed from *cis*-3 undergoes ring-closure to form a stabilized radical that results in an anomalously small BDE value of 51.4 kcal mol^−1^. While the DFT value lies close to this, the prediction is an overestimate by more than 40 kcal mol^−1^. However, *trans*-3, which differs only by the configuration of the central C=C bond, has a BDE value of 88.0 kcal mol^−1^. Ring-closure cannot occur in this case. The BDE prediction lies close to this value and the failure for *cis*-3 can be attributed to the occurrence of radical cyclization.

In constructing the BDE-db database, we omitted reactions where a bond dissociation resulted in an unstable radical that further decomposed into smaller species. While G4 calculations (which use uB3LYP/6-31G(2df,p) geometries) suggest that O–H dissociation of a carbamic acid group (Fig. 4c), results in the spontaneous loss of CO_2_, M06-2X calculations result in a weakly-bound adduct with a N–C bond length of 1.63 Å. Relative to the G4 value, both DFT and ML predictions in this case are inaccurate.

Another scenario resulting in BDE prediction outliers arises from difficult-to-converge electronic structure calculations for strongly delocalized systems (Fig. 4d). The O–H BDE values for phenols **7** is predicted by ALFABET as 89.2 kcal mol^−1^, whereas the reference DFT value is much higher at 108.3 kcal mol^−1^. The G4 value is much closer to the predicted BDE and suggest that in this case, it is the DFT value that is erroneous. Indeed, phenolic O–H bonds of neighboring molecules in the database have similar BDEs to the predicted value and further indicate that the DFT result is the outlier (Fig. 5b). The overestimate by DFT results from the convergence of open-shell structures to an incorrect electronic state. We found this was sensitive to the input structure used for geometry optimization and difficult to filter automatically (calculations are fully converged with a stable wavefunction) without prior knowledge of an expected BDE value.

In general, the most egregious ML-DFT prediction errors arise for conformations or electronic structures atypical with respect to the rest of the training database. Inspection of neighboring BDE values is therefore a qualitative method of determining whether a given BDE prediction is trustworthy: BDEs with several, similar neighbors with consistent BDEs lends additional confidence that a prediction is valid. The ALFABET webtool therefore includes the option to search for neighboring bonds from the training dataset. Using 3D features as inputs to the ML model might alleviate some of these prediction errors, although this would increase the computational cost of the ML predictions (as 3D coordinates would be required to generate predictions) and the possibility would remain of passing sub-optimal 3D inputs to the ML model and generating correspondingly poor DFT predictions. Additional filtering of DFT results might allow more accurate ALFABET predictions. However, ML prediction methods will likely never be able to appropriately predict the results of medium- to long-range intramolecular interactions without sufficient training examples.

### Application to bond dissociation in large molecules

**Fig. 6:**
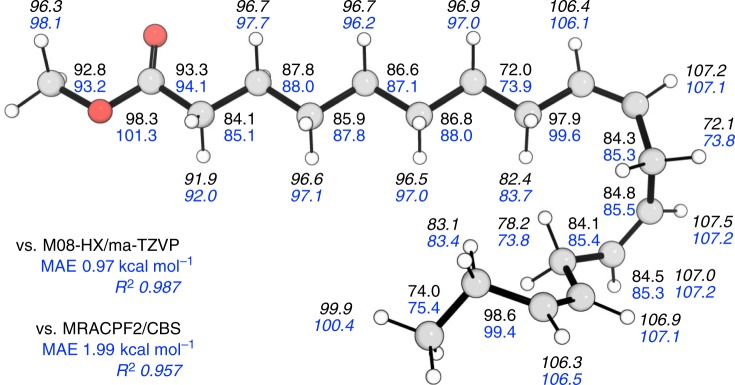
Applying the ML approach to BDE prediction in a large organic molecule. Comparison of BDE values (kcal mol^−1^) of the C–C, C–O, and C–H (italicized) bonds in methyl linolenate. ML values are in blue and M08-HX/ma-TZVP values are in black.

We used ALFABET to predict the C–C, C–O, and C–H bonds in methyl linolenate, an unsaturated fatty acid methyl ester found in biodiesel (Fig. 6). BDE values of biodiesel molecules are difficult to obtain experimentally and computational estimates are important for characterizing combustion chemistry, particularly the initial stages of pyrolysis. DFT BDE values have been obtained previously for methyl linolenate, in addition to multireference averaged coupled-pair functional (MRACPF2) values, which due to the large molecular size, were estimated using small surrogate models. The presence of C(sp^3^)–H, C(sp^2^)–H, C(sp^3^)–O, C(sp^3^)–C(sp^3^), and C(sp^3^)–C(sp^2^) bond types and carbonyl and olefin functional groups provides a good opportunity to test model performance. Pleasingly, our model provides BDE values very close to M08-HX/ma-TZVP (MAE of 0.97 kcal mol^−1^, R^2^ of 0.987^46^) and MRACPF2/CBS (MAE of 1.99 kcal mol^−1^, R^2^ of 0.957^42^), across 33 single bonds ranging in strengths by 34 kcal mol^−1^. The BDE values of weaker C–C and C–H bonds α-to the carbonyl and in allylic (and doubly-allylic) positions, along with those of stronger C(sp^2^)–C and C(sp^2^)–H bonds are all correctly described. This prediction, taking less than a second to complete, demonstrates the utility and accuracy of ALFABET for BDE prediction of larger, flexible hydrocarbons that are challenging to study by DFT and impossible for ab initio methods.

### Application to prediction of major sites of drug oxidation

The main advantage of the proposed method is that, due to its computational speed, it can be used in forward screening applications where DFT calculations would be infeasible. We therefore demonstrate the method’s applicability to two design challenges where BDEs play an important role in determining a molecule’s suitability. The first application is the pharmaceutical development of drug molecules, where predicting how a compound is likely to be metabolized can reduce failure rates in clinical trials^47^. Many xenobiotics are degraded by the cytochrome P450 enzyme, where the site of metabolism has been shown to correlate with the weakest C–H bond in the molecule^9^.

**Fig. 7:**
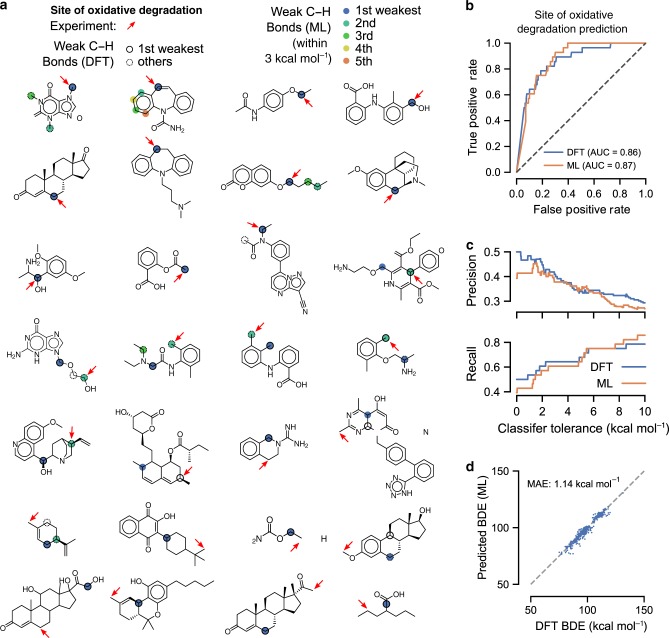
Application of ALFABET to predict site of oxidative degradation. **a** Structures of many of drug molecules where the site of oxidative degradation is known. Arrows indicate the experimentally determined breaking bond, while colors and circles indicate weakest bonds determined by ML and DFT, respectively. ML-predicted weakest bonds identify the experimental site in 11 out of the 28 molecules. **b** ROC curve for classifiers that predict the metabolic site through BDEs generated through ML or DFT. Both approaches yield similar performance. **c** Precision and recall of classifiers based on bond strengths calculated via DFT and ML approaches. Potential metabolic sites included all C–H bonds within a given energy from a molecule’s minimum. **d** Accuracy of the ML method in predicting BDEs for 82 large, drug-like molecules.

Calculation of C–H BDEs in drug screening, however, is a computationally expensive task, and we thus determined whether ALFABET demonstrates similar accuracy to a DFT-based calculation approach. We constructed a database of 28 drugs and their sites of oxidative degradation^8,9,48–51^. Drugs considered ranged in size from 6 to 32 heavy atoms. DFT calculations were then performed to determine the BDEs of all C–H bonds, and BDEs were also predicted using the developed GNN (Fig. 7a).

We then developed a site of metabolism classifier using the calculated BDEs. The weakest bonds in the molecule, within a certain energy tolerance, were predicted as possible targets for oxidation. The accuracy of the classifier, for BDEs derived both from DFT and from ALFABET, were quantified using a receiver operating characteristic (ROC) curve, Fig. 7b. This curve plots the true positive rate versus the false positive rate as the classifier tolerance is adjusted. The area under the curve (AUC) of the ROC curve thus represents a quantitative measure of the classifier’s performance, ranging from 0.5 (random guessing) to 1.0 (perfect predictions). The AUC for the DFT and ML-based classifiers was 0.86 and 0.87, respectively, indicating that the developed GNN is as accurate as DFT-based methods for predicting the site of metabolism, while requiring a fraction of the computational cost. In addition to an ROC curve, we also calculate precision and recall statistics for classifiers based on both DFT and ML bond strengths (Fig. 7c). Higher precision values indicate that the site of metabolism is present among only a few flagged candidate locations, while high recall values indicate the metabolic sites for most drugs are included among the predicted candidates. DFT-derived bond strengths appear yield a slightly higher maximum precision for tolerances <1 kcal mol^−1^, which likely represents the additional uncertainty imposed by the ML prediction. However, beyond this threshold precision and recall curves for both DFT and ML-derived bond strengths are similar, despite the substantially lower computational cost of ML. We note that our suggestions for the site of drug oxidation are only based on weakest bonds that do not explicitly account for accessibility of sites to the enzyme. These predictions could be further enhanced by incorporating accessibilities scores^52,53^.

To verify that ALFABET predictions are accurate for BDEs of drug molecules much larger than those used to construct the training set, DFT calculations then performed for 82 top-selling drug molecules^54^. These molecules ranged in size between 8 and 34 heavy atoms. Only H-atom BDEs were considered, resulting in 748 unique bonds broken. Despite only being trained on smaller molecules, the GNN successfully predicts the BDEs for much larger species, resulting in a MAE of 1.14 kcal mol^−1^ (Fig. 7d).

### Predicting combustion mechanisms from weakest bonds

In addition to metabolite decomposition, BDEs are essential in determining predominant combustion kinetic mechanisms. We next applied ALFABET to construct a mechanistically-inspired model of soot formation during combustion of new fuel chemistries. The yield sooting index (YSI) is an experimental measurement of the amount of soot a substance forms during combustion in a test flame^55,56^, and is an important parameter to consider during selection of potential fuel blendstocks^57^. While methods to predict YSI quickly from molecular structure exist^56,58^, these models do not leverage recent mechanistic understandings of how soot formation proceeds. Specifically, formation and growth of polyaromatic hydrocarbons (PAHs), the main component of particulate matter, is governed by the recombination of radicals formed in the combustion process.

**Fig. 8:**
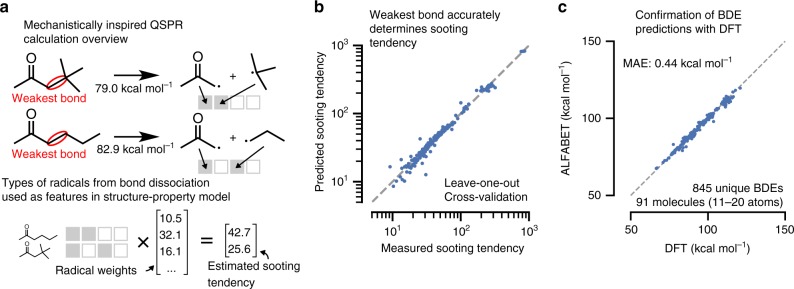
Development of a model for sooting tendency based on fast BDE calculation. **a** Overview of the QSPR approach. ALFABET predictions are used to determine a molecules weakest bond, which identifies the radicals used as features in the QSPR. **b** Results of the QSPR model under leave-one-out cross-validation. The model achieves a superior accuracy to a previous group-contribution method. **c** Confirmation of the predictions for molecules larger than those included in the training set.

In this study, we use our newly developed ML approach to predict the weakest bond in each of a set of 217 different fuel molecules with measured YSI values. The identities of the two radicals that form are then used to construct a QSPR model to predict soot formation. Instead of a series of descriptors or functional groups, each molecule was represented by only two parameters: one for each of the two radicals formed during cleaving of the weakest bond. These parameters are shared between molecules that decompose to form identical radicals (Fig. 8a). Molecules were chosen such that each radical was the result of at least two molecule decompositions.

We performed a leave-one-out cross-validation to determine the ability of the model to predict YSI for unseen molecules. In each cross-validation fold, a single compound was removed from the dataset and a weighted least-squares regression (with data weighted by their experimental uncertainty) was performed on the remainder of the data. Fitted radical weights are then used to predict the YSI of the held-out molecule. The cross-validated predictive accuracy of the new model, based on ALFABET predictions, achieves a weighted least-squares loss less than half that of a recently developed group-contribution model on the same dataset (Fig. 8b)^56^. These results demonstrate that AFLABET predictions can improve forward screening approaches in which bond energy is an important parameter.

We further verified that ALFABET is accurate for larger molecules outside the training set considered in this application. For the 91 molecules with YSI measurements and between 11 and 20 heavy atoms, DFT calculations were performed to confirm the predicted BDEs. The resulting prediction error was even lower than for the withheld test set predictions (Fig. 8c), demonstrating the ability of the model to scale to larger molecules.

## Discussion

In this study, we have developed a ML prediction tool to quickly calculate homolytic BDEs for organic molecules containing C, H, O, and N atoms, at an accuracy comparable with state-of-the-art DFT approaches. An interface for the developed prediction tool is available online at https://ml.nrel.gov/bde. Because BDEs are intrinsic properties of covalently bonded molecules, their relative strengths are important parameters in a wide range of chemical studies. We therefore expect our tool to enable high-throughput and accurate development of novel compounds for applications where elemental compositions are restricted to C, H, N, and O atoms and critical properties are determined by the strengths of single, non-ring bonds. Beyond the application areas to drug design and combustion pathways considered in this paper, we expect our tool to be useful in understanding polymer thermal stability, lignin depolymerization pathways, explosives, and high-performance energy-related materials. Future work will expand the training database to include other elements, bond types, and bonds in rings. As has been shown in a recent study, transfer learning may also permit improved accuracies through the incorporation of BDEs from well-curated experimental results^59^. While we have shown that high-accuracy CCSD(T) do not substantially improve accuracy over the chosen M06-2X method, databases of experimental bond dissociation energies do exist^33^. However, careful selection and fitting of experimental data will be required, as experimental BDEs measurements are biased toward the weakest bonds a molecule and sometimes have high uncertainty. More broadly, this study demonstrates the potential for deep learning techniques to accelerate quantum mechanical investigations where high-throughput computations are possible but time-consuming. Future work will look to expand these approaches to transition state structures.

## Methods

### Computational details for calculating homolytic BDEs

To sample radical conformations, H atoms were added to radical centers prior to MMFF structure optimization and removed afterward. MMFF94s performs well in conformational and non-covalent benchmarks involving neutral, closed-shell molecules^60^, however, it was not parametrized for radicals^42^. Unrestricted Kohn–Sham DFT calculations of radicals were carried out with careful consideration of electronic structures because M06-2X showed less accurate results in some aromatic radicals^61,62^. Specifically, spatial and spin symmetry of orbitals were broken by using the initial guess of mixed HOMO-LUMO with assuming no point-group symmetry of the structure. The stability of wavefunctions was also analyzed to confirm that the most stable electronic state had been found^63^. Convergence to the wrong electronic state occurred most frequently for aromatic radicals. Gaussian 16^64^ was used for all DFT calculations with a default ultra-fine grid for all numerical integration and for the G4 calculations to analyze outliers. DLPNO-CCSD(T) calculations were carried out with ORCA 4.0 as a single-point energy correction to the B3LYP-D3/6-31G(d) enthalpy using optimized geometries from B3LYP-D3/6-31G(d)^39^.

All optimizations were checked for convergence to an energy minimum, which included checking for proper termination flags from Gaussian and ensuring the resulting structure had no imaginary vibrational frequencies. In addition, we verified that the molecule did not decompose into separate molecules during the Gaussian optimization by ensuring that all bond lengths (expected from the Lewis structure) were <0.4 Å plus the sum of the covalent radii of the participating atoms. Finally, statistical tests on the completed database were used to screen for molecules with abnormally large enthalpies. For a given chemical formula (i.e., elemental composition), a linear model was used to predict overall molecule enthalpy. If residuals from this linear fit were >3 inner-quartile ranges from the predicted enthalpy, the molecule was discarded. This step removed a handful of high-energy, hypothetical molecules or ones that converged to unreasonable geometries. The BDE-db dataset has been published in an open-source database available on Figshare^65^.

### Graph neural network development

Determining the optimal inputs and structure to the GNN developed in this study was an iterative process in order to find one that yielded the lowest validation error. Nodes and edges were assigned to independent classes depending on a number of features. For nodes, unique classes were assigned based on an atom’s symbol, chirality tag, aromaticity, presence in ring (3, 4, 5, or ≥6), number of neighbors, and number of neighbor H’s. Edge classes were assigned based on the start atom symbol, end atom symbol, and presence of the bond in ring (3, 4, 5, or ≥ 6). The edge interaction network and atom state updating layers from Jorgensen et al.^43^ were simplified by removing layers until losses began to increase, and residual connections were added to the end of each message passing layers while batch normalization layers^66^ were added to the beginning of each message passing layer. The number of message passing layers was varied between 2 and 12, with validation losses not decreasing after six layers. Since the number of atoms for molecules in the training set was capped at nine, this allows messages to traverse the entire molecule except in a few select cases.

The loss function optimized the mean absolute error of all BDEs in the molecule, masking bonds for which DFT values were not available. Since edges in the model are directional, each bond has two corresponding edge states. During training, the BDE prediction of each directional edge is separately scored, while at test time the BDE prediction from both edges is averaged. The model was trained for 500 epochs using a batch size of 128 molecules with the ADAM optimizer using a learning rate of 1E−3 and a decay rate of 1E−5.

### GNN implementation

GNN models were implemented using the Python nfp library (https://github.com/nrel/nfp), which provides extensions to the Keras deep learning framework for modeling graph-valued systems. Models were trained using a single Nvidia Tesla V100 GPU for ~10–12 h.

### Calculating neighboring bonds

Intermediate layers in the GNN could be used to search for similar bonds in the DFT database for a given query bond. Embedding vectors for all bonds with calculated BDE values were generated from the output of the final message passing layer, a 128-dimensional vector. For computational efficiency, these vectors were reduced to a 10-dimensional vector through a principal component analysis (PCA). A nearest-neighbors search was then used to find the 10 closest bonds in the BDE-db database. The scikit-learn library^67^ was used to perform the PCA and nearest-neighbors searches.

## Supplementary information

Supplementary Information

Description of Additional Supplementary Files

Supplementary Data 1

Supplementary Data 2

## Data Availability

The datasets generated and/or analyzed during the current study are available on figshare with the identifier 10.6084/m9.figshare.10248932.

## Code availability

Weights for the final trained model and python scripts to generate predictions for new molecules has been made available through a Github repository (https://github.com/NREL/alfabet). Python scripts to train the model and Jupyter notebooks to create the figures in the paper are available at https://github.com/pstjohn/bde_model_methods.
