# Publisher Correction: Prediction of organic homolytic bond dissociation enthalpies at near chemical accuracy with sub-second computational cost

**DOI:** 10.1038/s41467-020-16706-7

**Published:** 2020-06-11

**Authors:** Peter C. St. John, Yanfei Guan, Yeonjoon Kim, Seonah Kim, Robert S. Paton

**Affiliations:** 1 Biosciences Center, National Renewable Energy Laboratory, 15103 Denver West Parkway, Golden, CO 80401 USA; 2 Department of Chemistry, Colorado State University, Fort Collins, Colorado 80523 USA; 3 Chemical Research Laboratory, University of Oxford, Mansfield Road, Oxford, OX1 3TA UK; 4 Present Address: Department of Chemical Engineering, Massachusetts Institute of Technology, 77 Massachusetts Ave., Cambridge, MA 02139 USA

**Keywords:** Cheminformatics, Thermodynamics, Computational chemistry

Correction to: *Nature Communications* 10.1038/s41467-020-16201-z, published online 11 May 2020.

The original version of this Article contained some errors in Fig. 7, in which there were four structures where one of the atoms was inadvertently displaced to the right of the structures.

The correct version of Fig. 7 is



which replaces the previous incorrect version from the original pdf of the paper.

This has been corrected in the PDF and HTML versions of the Article.

