# Supplementary material for: Prediction of organic homolytic bond dissociation enthalpies at near chemical accuracy with sub-second computational cost
Source: Nat Commun. 2020 May 11;11:2328. doi: 10.1038/s41467-020-16201-z (PMC7214445; doi:10.1038/s41467-020-16201-z)
Supplement: Supplementary file 1 — Supplementary Information [file 41467_2020_16201_MOESM1_ESM.pdf]

Supporting Information

Prediction of organic homolytic bond dissociation enthalpies at near chemical accuracy with sub-second computational cost

St. John et al.

Supplementary Figures

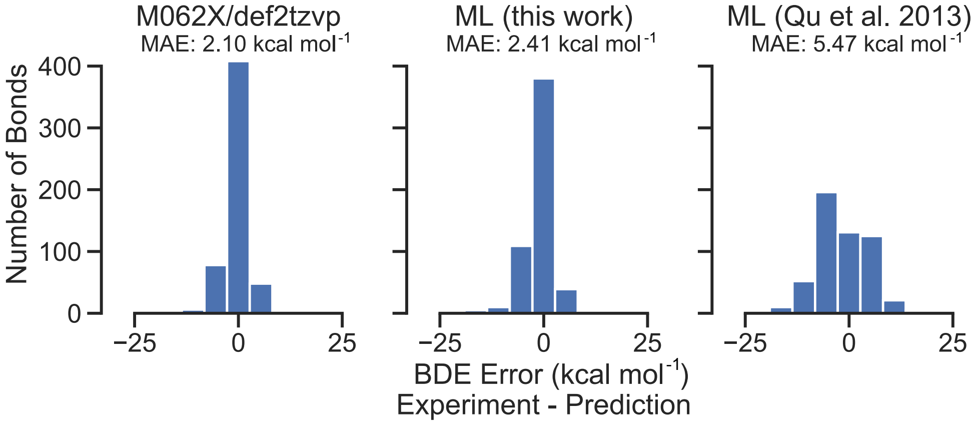

Supplementary Figure 1: Comparison against previous BDE prediction methods. DFT calculations and predictions from the ALFABET model are compared against those using the Neural Network model of Qu *et al.* 2013.[28]

Supplementary Tables

Supplementary Table 1: Calculated Enthalpies for Error Analysis Calculations (Figure 4). All enthalpies are in Hartrees.

	**Molecule**	**Radical**	**ΔH$_f$ (mol, DFT)**	**ΔH$_f$ (mol, G4)**	**ΔH$_f$ (rad, DFT)**	**ΔH$_f$ (rad, G4)**
**A**	O=C1CCCC/C1=C/O	[O]/C=C1/CCCCC1=O	-423.02388	1a: -423.002035<br>1b: -422.983174	-422.37628	2dot: -422.358274
**B**	C/C=C(C)/C=C\C(=O)OC	C/C=C(C)/C=C\C([O])=O	-462.2693	cis-3: -462.256891<br>trans-3: -462.264775	-422.39872	cis-4dot: -462.264775<br>trans-4dot: -422.328661
**C**	NC(=O)CCN(N)C(=O)O	NC(=O)CCN(N)C([O])=O	-547.63572	5: -547.576587	-546.99392	6dot: -546.958135<br>*(includes CO_2)*
**D**	Nc1cc(N)cc(O)c1	Nc1cc(N)cc([O])c1	-418.03083	7: -417.999734	-417.36249	8dot: -417.363114
