# Supplementary material for: Prediction of organic homolytic bond dissociation enthalpies at near chemical accuracy with sub-second computational cost
Source: Nat Commun. 2020 May 11;11:2328. doi: 10.1038/s41467-020-16201-z (PMC7214445; doi:10.1038/s41467-020-16201-z)
Supplement: Supplementary file 2 — Description of Additional Supplementary Files [file 41467_2020_16201_MOESM2_ESM.docx]

Description of Additional Supplementary Files

File name: Supplementary Movie 1

Description: 3D reconstruction of a droplet replica cluster at the centre of a network. 360° rotation and horizontal cross-sections of the 3D reconstruction of a fluorescent droplet replica cluster composed of 14 droplets arranged in a hcp lattice (relative to Fig. 5 and Supplementary Fig. 9a-k).

File name: Supplementary Movie 2

Description: 3D reconstruction of a droplet replica cluster at the top of a network. 360° rotation and horizontal cross-sections of the 3D reconstruction of a fluorescent droplet replica cluster composed of 6 droplets on the top of a network (Supplementary Fig. 5l-p).
